# Supplementary material for: A Pilot Study on Pharmacists’ Knowledge, Attitudes and Practices towards Medication Dysphagia via Asynchronous Online Focus Group Discussion
Source: Int J Environ Res Public Health. 2023 Feb 6;20(4):2858. doi: 10.3390/ijerph20042858 (PMC9956395; doi:10.3390/ijerph20042858)
Supplement: Supplementary file 1 [file ijerph-20-02858-s001.zip › ijerph-2092258-supplementary.pdf]

Table S1. Table of Comparison of Platforms Evaluated. *Initial cut-off criteria included Platform-provided Hosting and Browser-based. Key criteria for further evaluation included Asynchronous Discussion, Participant Anonymity, Controlled User Access (by password-controlled or invite-only access), Deletion of Data on Request, and Data Collected Not Sold.*

Platform	**Platform-provided Hosting**	**Browser-based**	**Asynchronous Discussion**	**Anonymity**	**Controlled User Access**	**Deletion of Data on Request**	**Data Collected Not Sold**	**Threading and Nested Replies**	**Download-able Responses**
FocusGroupIt	✓	✓	✓	✓ *	✓	✓	✓	✓	✓
NowComment	✓	✓	✓	✓ ^	✓	✓	✓	✓	---
Discourse	✓	✓	✓	✓	✓	✓	✓	✓	---
Ning	✓	✓	✓	---	✓	✓	✓	---	---
Microsoft Teams	✓	✓ (App optional)	✓	✗	✓	✓	✓	✓	---
Collabito	✓	✓	✓	✗	---	✓	✓	✓	✓
BackChannel Chat	✓	✓	✗	✓	✓	---	✓	---	✗
ListServ	✓	✓	✓	✗	✗	---	---	✓	✓
VBulletin Cloud	✓	---	✓	---	✓	---	✓	✓	✓
Itracks	---	✗ (App required)							
Invision	✗								
FluxBB	✗								
phpBB	✗								

* Pseudonym (manually) with free plan; option with paid plan

^ Pseudonym (manually)
